# Mental Health Problems Among School-Aged Children After School Reopening: A Cross-Sectional Study During the COVID-19 Post-pandemic in East China

**DOI:** 10.3389/fpsyg.2021.773134

**Published:** 2021-11-11

**Authors:** Jingyi Wang, Yingying Wang, Haijiang Lin, Xiaoxiao Chen, Hao Wang, Hongbiao Liang, Xiaoqin Guo, Chaowei Fu

**Affiliations:** ^1^Key Laboratory of Public Health Safety, NHC Key Laboratory of Health Technology Assessment, School of Public Health, Fudan University, Shanghai, China; ^2^Taizhou City Center for Disease Prevention and Control, Taizhou, China; ^3^Songjiang District Center for Disease Prevention and Control, Shanghai, China

**Keywords:** mental health, psychological stressors, children, school reopening, COVID-19

## Abstract

**Background:** Most studies on mental health problems caused by COVID-19 crisis in children were limited to the period of home quarantine. It remained unclear what adverse impact of the psychosocial stressors caused by school reopening, as well as the transitions in daily activities and social interactions had on mental health in children.

**Methods:** A total of 6400 students in primary schools were enrolled in a cross-sectional study conducted in East China, between June 26 and July 6, 2020, when schools reopened. Children’s mental health status was assessed by the parent version of Strengths and Difficulties Questionnaire (SDQ). Ultimately, data on a total of 6017 children with completed information on mental health, psychosocial stressors, daily activities, and social interactions were eligible for analysis. The associations of mental health with psychosocial stressors, daily activities, and social interactions were determined by ordinal logistic regression models. Stratified analyses were conducted according to grade, gender, school level, area, and caregiver–child relationship to further observe the effects of stressors on mental status.

**Results:** The prevalence of borderline, moderately abnormal, and prominently abnormal scores were 7.16, 3.34, and 1.96% for total difficulties, and 13.83, 13.45, and 17.85% for prosocial behavior, respectively. Children with psychological stressors had a significantly higher risk of being in a worse category of mental health status, with the maximum adjusted OR of 7.90 (95% CI 3.33–18.75) in those definitely afraid of inadaptation to study and life styles. Time used in home work and computer games was positively related to mental health problems, while physical exercises and frequency of communication with others was negatively related. The effects of psychological stressors on total difficulties were more evident in middle-high grade students (OR = 7.52, 95% CI 4.16–8.61), boys (OR = 6.95, 95% CI 4.83–8.55), those who lived in Taizhou (OR = 7.62, 95% CI 4.72–8.61) and with poor caregiver–child relationship (OR = 7.79, 95% CI 2.26–8.65).

**Conclusion:** Emotional and behavioral difficulties, especially less prosocial behavior, were prevalent in primary school children after schools reopened. The Chinese government, communities, schools, and families need to provide more effective support for students’ transition back into the school building and address emotional and behavioral problems for children with difficulties.

## Introduction

The pandemic of COVID-19, which was caused by the severe acute respiratory syndrome coronavirus 2 (SARS-CoV-2) (Zhu et al., 2020), has been declared as an international public health emergency by the World Health Organization (WHO), 2021. The Chinese government had implemented a nationwide school closure in response to the outbreak in order to protect the students through reducing contacts with others since early February, 2020. More than 220 million children and adolescents in China were restricted to their homes, including 180 million primary students and 47 million preschool children (CCTV News, 2021). The dates of primary schools reopening across provinces in China were inconsistent, progressively from March 23, 2020, in Xinjiang Province (People’s Daily Online, 2021b), to June 15, 2020, in Hebei Province (People’s Daily Online, 2021a), according to the local epidemic situation and prevention and control strategies.

This emergency measure has brought remarkable benefit of preventing the transmission of COVID-19 (Zhang et al., 2020), but children and adolescents, as the vulnerable groups, had been affected by the adverse impacts of prolonged COVID-19-related school closure and home confinement on physical and mental health (Saurabh and Ranjan, 2020; Xie et al., 2020; Li et al., 2021). They had encountered a series of difficult transitions in daily activities (e.g., irregular rest timetable, lack of personal space at home, less outdoor activities, and more time used on electronic equipment), and social interactions (e.g., more dependence on social media instead of face-to-face contact with peers, teachers, and others) (Ezpeleta et al., 2020). Some mental health threats were generated during the transitional period, such as loneliness (Guessoum et al., 2020) and intensified caregiver–child relationship (Cusinato et al., 2020). A study investigated the psychological responses of children to pandemic disasters and reported that children who experienced isolation or quarantine were more likely to meet the clinical cutoff score for post-traumatic stress disorder (PTSD) than those who did not (30 vs. 1.1%) (Sprang and Silman, 2013). However, it remains unclear what mental status was in children after schools reopened and what influence the transitions in daily activities and social interactions had on mental health in children during COVID-19 post-pandemic period.

Furthermore, the dates of school reopening had been repeatedly postponed, although all the subjects were taught online, including language, math, science, even gym, and art (Wang et al., 2020). As a result of lack of school rhythm and routines, group study atmosphere and effective monitoring, students may fail to adapt to online learning at home, lose interest in studying, and have diminished attention and academic performance (Zhao et al., 2020). After the lockdown period ended and schools reopened, children returned to a new studying lifestyle with restrictive measures for COVID-19. They had to wear facemasks, adhere to hand hygiene, and keep physical distance. The joint effects of the disruption in normal classroom schooling and concerns of social distancing could be considered as psychosocial stressors which may lead to maladjustment and make children vulnerable to mental health problems, including fear (Jiao et al., 2020), anxiety (Duan et al., 2020), and depression (Zhou et al., 2020). Therefore, further studies are warranted to investigate the degrees of psychological stressors and their adverse effects on mental health in children when schools reopened.

Although the knowledge regarding children’s reactions to this unprecedented COVID-19 crisis has been expanded, the majority of studies were limited to the duration of school closure and home quarantine (Guessoum et al., 2020; Khan et al., 2020; Xie et al., 2020), and further observations after school reopening should be continued. The feasibility and effectiveness of school closure policies on mitigating the epidemics of infectious diseases were well-known (Fumanelli et al., 2016), but it remains unclear what the negative impact of long-term school closures have on psychological and behavioral problems in children, especially when they return to schools and face school life again. Therefore, the aim of this study was to investigate the prevalence of mental health problems among primary school students after schools reopened. We also aimed to evaluate the associations between psychological stressors, daily activities, social interactions, and mental health status, in order to provide suggestions for mental health promotion in school-aged children.

## Materials and Methods

### Study Design and Subject Selection

**FIGURE 1:**
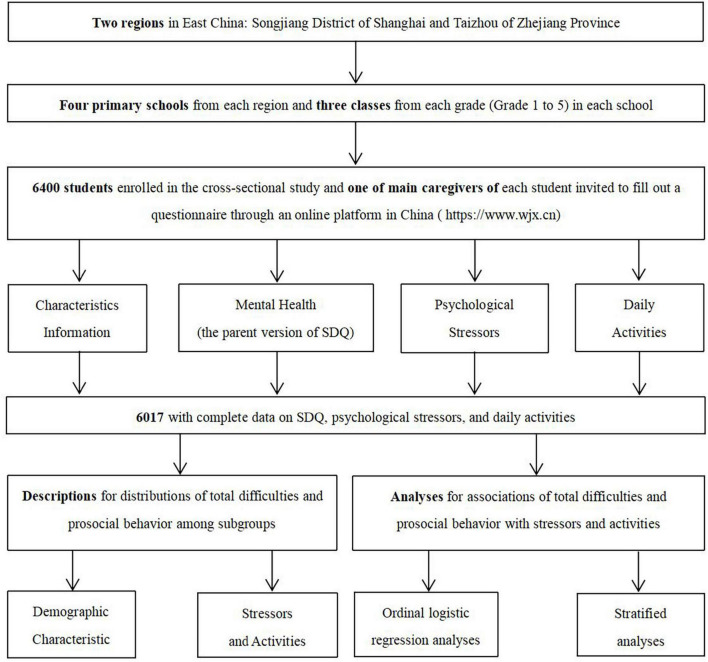
Flow chart.

A cross-sectional study was conducted by using a cluster sampling method among primary school students in Songjiang District of Shanghai and Taizhou of Zhejiang Province, with different levels of education and economic development in East China, between June 26 and July 6, 2020, when primary schools in these regions have been reopened. Primary schools in Shanghai reopened on May 18, 2020 for Grade 4 and 5 and June 2, 2020 for Grade 1–3, while primary schools in Taizhou reopened on April 20, 2020 for Grade 4–6 and April 26, 2020 for Grade 1–3. Four primary schools of different characteristics, including key schools and non-key schools, were randomly sampled from each region, and three classes from each grade (Grade 1–5) were randomly selected in each school. A total of 6400 students from these classes were enrolled in the study after excluding 204 individuals who refused to participate. One of main caregivers of each student was invited to fill out a questionnaire through an online platform in China^1^ to report information about their child. Ultimately, data on a total of 6017 students with complete information on scores of the parent version of the Strengths and Difficulties Questionnaire (SDQ), psychological stressors, daily activities, and social communication ways were used in statistical analyses after removing those with invalid questionnaires. The response rate was 94.02%. A flow chart was shown in Figure 1. Informed written consents were obtained from all subjects and their caregivers, and this study was approved by the ethical review board of the School of Public Health of Fudan University (IRB#2020040817).

### Information Collection for Sociodemographic Characteristics

Information on characteristics of children was collected, including age, grade, gender, school level (“non-key school” and “key school.” Teaching equipment, students’ academic performance, and quality of teachers in key schools are generally better than those in non-key schools), child medical conditions (hypertension, diabetes, cardiovascular diseases, pulmonary diseases, cancers, other chronic physical diseases, anxiety, depression, other psychiatric illnesses, attention-deficit disorder/attention-deficit hyperactivity disorder, autism spectrum disorder, lack of outdoor activities due to disability and any other disability), knowledge, and precaution (preventive behaviors) levels on COVID-19 (score 1–7). Information on characteristics of their families was also recorded, including area, number of household rooms, family economic status (“low,” “middle,” and “high”), caregiver education level (“primary school or below,” “middle or high school,” and “college or above”), and caregiver medical conditions (pregnancy, hypertension, diabetes, cardiovascular diseases, pulmonary diseases, cancers, other chronic physical diseases, anxiety, depression, other psychiatric illnesses, lack of outdoor activities due to disability and any other disability), as well as caregiver–child relationship (the question was “Do you agree with the statement that you have a good relationship with your child?” The corresponding responses were “Strongly disagree,” “Disagree,” “Agree,” and “Strongly agree.” Finally, the responses were categorized into two groups: “poor” and “good”) (Table 1).

**TABLE 1:** Distributions of total difficulties scores and prosocial behavior scores by different characteristics.

	***n* (%)^a^**	**Strengths and Difficulties Questionnaire (SDQ)**									
		**Total difficulties**					**Prosocial behavior**				
		**Normal (0–13)**	**Borderline (14–16)**	**Moderately abnormal (17–19)**	**Prominently abnormal (20–40)**	***P*-value**	**Normal (8–10)**	**Borderline (7)**	**Moderately abnormal (6)**	**Prominently abnormal (0–5)**	***P*-value**
**Total (%)**	6017 (100)	5267 (87.5)	431 (7.2)	201 (3.3)	118 (2)		3302 (54.9)	832 (13.8)	809 (13.5)	1074 (17.9)	
**Child characteristics**											
**Grade**						0.265					0.043
Grade 1	1269 (21.1)	1127 (88.8)	91 (7.2)	35 (2.8)	16 (1.3)		704 (55.5)	183 (14.4)	182 (14.3)	200 (15.8)	
Grade 2	1200 (19.9)	1045 (87.1)	87 (7.3)	37 (3.1)	31 (2.6)		643 (53.6)	175 (14.6)	177 (14.8)	205 (17.1)	
Grade 3	1262 (21)	1086 (86.1)	104 (8.2)	44 (3.5)	28 (2.2)		685 (54.3)	159 (12.6)	166 (13.2)	252 (20)	
Grade 4	1233 (20.5)	1077 (87.4)	85 (6.9)	50 (4.1)	21 (1.7)		708 (57.4)	167 (13.5)	157 (12.7)	201 (16.3)	
Grade 5	1053 (17.5)	932 (88.5)	64 (6.1)	35 (3.3)	22 (2.1)		562 (53.4)	148 (14.1)	127 (12.1)	216 (20.5)	
**Gender**						<0.001					<0.001
Boy	3287 (54.6)	2810 (85.5)	267 (8.1)	130 (4)	80 (2.4)		1727 (52.5)	453 (13.8)	466 (14.2)	641 (19.5)	
Girl	2730 (45.4)	2457 (90)	164 (6)	71 (2.6)	38 (1.4)		1575 (57.7)	379 (13.9)	343 (12.6)	433 (15.9)	
**School level**						0.506					<0.001
Non-key school	4924 (81.8)	4296 (87.3)	363 (7.4)	168 (3.4)	97 (2)		2635 (53.5)	694 (14.1)	691 (14)	904 (18.4)	
Key school	1093 (18.2)	971 (88.8)	68 (6.2)	33 (3)	21 (1.9)		667 (61)	138 (12.6)	118 (10.8)	170 (15.6)	
**Child medical conditions**						<0.001					0.008
No	5943 (98.8)	5224 (87.9)	422 (7.1)	191 (3.2)	106 (1.8)		3272 (55.1)	821 (13.8)	800 (13.5)	1050 (17.7)	
Yes	74 (1.2)	43 (58.1)	9 (12.2)	10 (13.5)	12 (16.2)		304 (40.5)	11 (14.9)	9 (12.2)	24 (32.4)	
**Knowledge levels on COVID-19**						<0.001					<0.001
Rare (score 1–2)	825 (13.7)	637 (77.2)	105 (12.7)	50 (6.1)	33 (4)		288 (34.9)	122 (14.8)	164 (19.9)	251 (30.4)	
Moderate (score 3–5)	3256 (54.1)	2847 (87.4)	234 (7.2)	114 (3.5)	61 (1.9)		1655 (50.8)	502 (15.4)	451 (13.9)	648 (19.9)	
Sufficient (score 6–7)	1936 (32.2)	1783 (92.1)	92 (4.8)	37 (1.9)	24 (1.2)		1359 (70.2)	208 (10.7)	194 (10)	175 (9)	
**Precaution levels on COVID-19**						<0.001					<0.001
Rare (score 1–2)	811 (1.4)	566 (69.1)	10 (12.4)	10 (12.4)	5 (6.2)		222 (27.2)	44 (4.9)	18 (22.2)	37 (45.7)	
Moderate (score 3–5)	827 (13.7)	665 (80.4)	88 (10.6)	45 (5.4)	29 (3.5)		283 (34.2)	115 (13.9)	160 (19.4)	269 (32.5)	
Sufficient (score 6–7)	5109 (84.9)	4546 (89)	333 (6.5)	146 (2.9)	84 (1.6)		2997 (58.7)	713 (14)	631 (12.4)	768 (15)	
**Family characteristics**											
**Area**						0.831					0.039
Taizhou	2652 (44.1)	2330 (87.9)	189 (7.1)	85 (3.2)	48 (1.8)		1404 (52.9)	394 (14.9)	372 (14)	482 (18.2)	
Shanghai	3365 (55.9)	2937 (87.3)	242 (7.2)	116 (3.4)	70 (2.1)		1898 (56.4)	438 (13)	437 (13)	592 (17.6)	
**Number of household rooms**						<0.001					<0.001
1 room	775 (12.9)	641 (82.7)	82 (10.6)	33 (4.3)	19 (2.5)		359 (46.3)	100 (12.9)	138 (17.8)	178 (23)	
2–3 rooms	3409 (56.7)	2991 (87.7)	240 (7)	107 (3.1)	71 (2.1)		1891 (55.5)	460 (13.5)	453 (13.3)	605 (17.8)	
≥4 rooms	1833 (30.5)	1635 (89.2)	109 (6)	61 (3.3)	28 (1.5)		1052 (57.4)	272 (14.8)	218 (11.9)	291 (15.9)	
**Family economic status**						<0.001					<0.001
Low	470 (7.8)	352 (74.9)	56 (11.9)	37 (7.9)	25 (5.3)		213 (45.3)	61 (13)	88 (18.7)	108 (23)	
Middle	4927 (81.9)	4346 (88.2)	344 (7)	152 (3.1)	85 (1.7)		2658 (54)	702 (14.3)	668 (13.6)	899 (18.3)	
High	620 (10.3)	569 (91.8)	31 (5)	12 (1.9)	81 (1.3)		431 (69.5)	69 (11.1)	53 (8.6)	67 (10.8)	
**Caregiver education level**						<0.001					<0.001
Primary school or below	415 (6.9)	331 (79.8)	51 (12.3)	15 (3.6)	18 (4.3)		219 (52.8)	48 (11.6)	50 (12.1)	98 (23.6)	
Middle or high school	3250 (54)	2808 (86.4)	260 (8)	120 (3.7)	62 (1.9)		1673 (51.5)	456 (14)	491 (15.1)	630 (19.4)	
College or above	2352 (39.1)	2128 (90.5)	120 (5.1)	66 (2.8)	38 (1.6)		1410 (60)	328 (14)	268 (11.4)	346 (14.7)	
**Caregiver medical conditions**						<0.001					0.214
No	5529 (91.9)	4879 (88.2)	381 (6.9)	169 (3.1)	100 (1.8)		3053 (55.2)	766 (13.9)	733 (13.3)	977 (17.7)	
Yes	488 (8.1)	388 (79.5)	50 (10.3)	32 (6.6)	18 (3.7)		249 (51)	66 (13.5)	76 (15.6)	97 (19.9)	
**Caregiver–child relationship**						<0.001					<0.001
Poor	308 (5.1)	212 (68.8)	43 (14)	26 (8.4)	27 (8.8)		131 (42.5)	40 (13)	41 (13.3)	96 (31.2)	
Well	5709 (94.9)	5055 (88.5)	388 (6.8)	175 (3.1)	91 (1.6)		3171 (55.5)	792 (13.9)	768 (13.5)	978 (17.1)	

*^a^The number and proportions of children within each category of sociodemographic characteristics.*

### Information Collection for Psychological Stressors, Daily Activities, and Social Interactions

Psychological stressors for children were obtained through a self-made questionnaire, regarding the degree to which a child was afraid of each situation after school reopened (1) going to school, (2) lagging behind in school lessons, (3) inadaptation to study and life styles, (4) keeping social distance with others, and (5) changes in relationship with schoolmates. The responses were defined into four levels (“completely not,” “slightly,” “moderately,” and “definitely”). Children’s daily activities and social interactions were also collected, in terms of length of time or frequency a child conducting each in the last week (1) online learning, (2) homework, (3) computer games, (4) physical exercises, (5) telephone or video calling, (6) text messaging or social media communication, and (7) meeting with friends or relatives. The responses were defined into three levels (“≤1,” “1–3,” and “>3 h” for the first three items, “none,” “≤30 min,” and “>30 min” for the fourth item, and “none,” “≤1 time,” and “>1 time” for the last three items) and were presented in Table 2.

**TABLE 2:** Distributions of total difficulties score and prosocial behavior score by psychological stressors, daily activities, and social interactions.

	***n* (%)^a^**	**Strengths and Difficulties Questionnaire (SDQ)**									
		**Total difficulties**					**Prosocial behavior**				
		**Normal (0–13)**	**Borderline (14–16)**	**Moderately abnormal (17–19)**	**Prominently abnormal (20–40)**	***P*-value**	**Normal (8–10)**	**Borderline (7)**	**Moderately abnormal (6)**	**Prominently abnormal (0–5)**	***P*-value**
**Total (%)**	6017 (7100)	5267 (87.5)	431 (7.2)	201 (3.3)	118 (2)		3302 (54.9)	832 (13.8)	809 (13.5)	1074 (17.8)	
**Psychological stressors^b^**											
**Going to school**						<0.001					<0.001
Completely not	3163 (52.6)	2949 (93.2)	138 (4.4)	50 (1.6)	26 (0.8)		2119 (67)	400 (12.7)	317 (10)	327 (10.3)	
Slightly	2169 (36.1)	1842 (84.9)	183 (8.4)	98 (4.5)	46 (2.1)		977 (45)	335 (15.4)	364 (16.8)	493 (22.7)	
Moderately	620 (10.3)	437 (70.5)	96 (15.5)	47 (7.6)	40 (6.5)		189 (30.5)	89 (14.4)	119 (19.2)	223 (36)	
Definitely	651 (1.1)	39 (60)	14 (21.5)	69 (9.2)	69 (9.2)		172 (26.2)	81 (12.3)	9 (13.9)	31 (47.7)	
**Lagging behind in school lessons**						<0.001					<0.001
Completely not	2256 (37.5)	2088 (92.6)	89 (4)	52 (2.3)	27 (1.2)		1370 (60.7)	290 (12.9)	272 (12.1)	324 (14.4)	
Slightly	2619 (43.5)	2279 (87)	214 (8.2)	88 (3.4)	38 (1.5)		1342 (51.2)	375 (14.3)	367 (14)	535 (20.4)	
Moderately	792 (13.2)	634 (80.1)	85 (10.7)	42 (5.3)	31 (3.9)		381 (48.1)	126 (15.9)	127 (16)	158 (20)	
Definitely	350 (5.8)	266 (76)	43 (12.3)	19 (5.4)	22 (6.3)		209 (59.7)	41 (11.7)	43 (12.3)	57 (16.3)	
**Inadaptation to study and life styles**						<0.001					<0.001
Completely not	3739 (62.1)	3486 (93.2)	156 (4.2)	65 (1.7)	32 (0.9)		2364 (63.2)	482 (12.9)	418 (11.2)	475 (12.7)	
Slightly	2093 (34.8)	1665 (79.6)	243 (11.6)	115 (5.5)	70 (3.3)		860 (41.1)	331 (15.8)	356 (17)	546 (26.1)	
Moderately	161 (2.7)	102 (63.4)	29 (18)	19 (11.8)	11 (6.8)		694 (42.9)	18 (11.2)	31 (19.3)	43 (26.7)	
Definitely	240 (0.4)	145 (58.3)	31 (12.5)	2 (8.3)	5 (20.8)		937 (37.5)	14 (4.2)	4 (16.7)	10 (41.7)	
**Keeping social distance with others**						<0.001					<0.001
Completely not	3264 (54.3)	2978 (91.2)	175 (5.4)	73 (2.2)	38 (1.2)		1943 (59.5)	462 (14.2)	375 (11.5)	484 (14.8)	
Slightly	2196 (36.5)	1839 (83.7)	202 (9.2)	101 (4.6)	54 (2.5)		1057 (48.1)	297 (13.5)	354 (16.1)	488 (22.2)	
Moderately	396 (6.6)	325 (82.1)	36 (9.1)	17 (4.3)	18 (4.6)		196 (49.5)	57 (14.4)	58 (14.7)	85 (21.5)	
Definitely	161 (2.7)	125 (77.6)	18 (11.2)	10 (6.2)	84 (5)		106 (65.8)	16 (9.9)	22 (13.7)	17 (10.6)	
**Changes in relationship with schoolmates**						<0.001					<0.001
Completely not	4164 (69.2)	3831 (92)	209 (5)	87 (2.1)	37 (0.9)		2488 (59.8)	548 (13.2)	495 (11.9)	633 (15.2)	
Slightly	1478 (24.6)	1160 (78.5)	178 (12)	89 (6)	51 (3.5)		617 (41.8)	244 (16.5)	255 (17.3)	362 (24.5)	
Moderately	247 (4.1)	176 (71.3)	30 (12.2)	18 (7.3)	23 (9.3)		116 (47)	33 (13.4)	39 (15.8)	59 (23.9)	
Definitely	128 (2.1)	100 (78.1)	14 (10.9)	75 (5.5)	75 (5.5)		816 (63.3)	75 (5.5)	20 (15.6)	20 (15.6)	
**Daily activities in the last week^c^**											
**Online learning**						0.231					0.059
<1 h/day	2902 (48.2)	2565 (88.4)	189 (6.5)	91 (3.1)	57 (2)		1583 (54.6)	416 (14.3)	406 (14)	497 (17.1)	
1–3 h/day	1464 (24.3)	1264 (86.3)	113 (7.7)	61 (4.2)	26 (1.8)		785 (53.6)	216 (14.8)	202 (13.8)	261 (17.8)	
>3 h/day	1625 (27)	1420 (87.4)	125 (7.7)	47 (2.9)	33 (2)		926 (57)	196 (12.1)	195 (12)	308 (19)	
**Homework**						<0.001					<0.001
<1 h/day	1446 (24)	1319 (91.2)	71 (4.9)	30 (2.1)	26 (1.8)		846 (58.5)	185 (12.8)	176 (12.2)	239 (16.5)	
1–3 h/day	3911 (65)	3461 (88.5)	277 (7.1)	118 (3)	55 (1.4)		2146 (54.9)	560 (14.3)	532 (13.6)	673 (17.2)	
>3 h/day	627 (10.4)	464 (6474)	78 (12.4)	50 (8)	35 (5.6)		295 (47.1)	81 (12.9)	95 (15.2)	156 (24.9)	
**Computer games**						<0.001					<0.001
None	2709 (45)	2467 (91.1)	148 (5.5)	59 (2.2)	35 (1.3)		1638 (60.5)	343 (12.7)	315 (11.6)	413 (15.3)	
=1 time/day	2438 (40.5)	2153 (88.3)	157 (6.4)	90 (3.7)	38 (1.6)		1298 (53.2)	359 (14.7)	347 (14.2)	434 (17.8)	
>1 time/day	870 (14.5)	647 (74.4)	126 (14.5)	52 (6)	45 (5.2)		366 (42.1)	130 (14.9)	147 (16.9)	227 (26.1)	
**Physical exercise**						<0.001					<0.001
None	375 (6.2)	252 (67.2)	61 (16.3)	27 (7.2)	35 (9.3)		133 (35.5)	49 (13.1)	63 (16.8)	130 (34.7)	
=30 min/day	3065 (50.9)	2642 (86.2)	248 (8.1)	117 (3.8)	58 (1.9)		1479 (48.3)	474 (15.5)	478 (15.6)	634 (20.7)	
>30 min/day	2577 (42.8)	2373 (92.1)	122 (4.7)	57 (2.2)	25 (1)		1690 (65.6)	309 (12)	268 (10.4)	310 (12)	
**Social interactions in the last week^c^**											
**Telephone or video calling communication**						<0.001					<0.001
None	1448 (24.1)	1207 (83.4)	122 (8.4)	68 (4.7)	51 (3.5)		697 (48.1)	189 (13.1)	216 (14.9)	346 (23.9)	
=1 time/day	3689 (61.3)	3283 (89)	242 (6.6)	112 (3)	52 (1.4)		2067 (56)	533 (14.5)	480 (13)	609 (16.5)	
>1 time/day	880 (14.6)	777 (88.3)	67 (7.6)	21 (2.4)	15 (1.7)		538 (61.1)	110 (12.5)	113 (12.8)	119 (13.5)	
**Text messaging or social media communication**						<0.001					<0.001
None	1912 (31.8)	1613 (84.4)	156 (8.2)	85 (4.5)	58 (3)		949 (49.6)	264 (13.8)	275 (14.4)	424 (22.2)	
=1 time/day	2852 (47.4)	2539 (89)	194 (6.8)	82 (2.9)	37 (1.3)		1599 (56.1)	406 (14.2)	375 (13.2)	472 (16.6)	
>1 time/day	1253 (20.8)	1115 (89)	81 (6.5)	34 (2.7)	23 (1.8)		754 (60.2)	162 (12.9)	159 (12.7)	178 (14.2)	
**Meeting with friends or relatives**						<0.001					<0.001
None	2423 (40.3)	2102 (86.8)	180 (7.4)	84 (3.5)	57 (2.4)		1283 (53)	329 (13.6)	320 (13.2)	491 (20.3)	
=1 time/day	2800 (46.5)	2498 (89.2)	168 (6)	91 (3.3)	43 (1.5)		1548 (55.3)	392 (14)	397 (14.2)	463 (16.5)	
>1 time/day	794 (13.2)	667 (84)	83 (10.5)	26 (3.3)	18 (2.3)		471 (59.3)	111 (14)	92 (11.6)	120 (15.1)	

*^*a*^The number and proportions of children within each category of psychological stressors, daily activities, and social interactions.*

*^*b*^The degree to which a child was afraid of each situation after school reopened.*

*^*c*^The length of time/frequency a child conducted each activity in the last week.*

### Assessments of Children’s Mental Health

The parent version of the SDQ was adopted to assess mental health of children according to caregivers’ observations (Stone et al., 2010). Five subscales included in SDQ were designed for evaluating emotional symptoms, conduct problems, hyperactivity/inattention, peer relationship problems, and prosocial behavior, respectively. Each subscale includes five items and each item is scored on a 3-point scale (0 = not true, 1 = somewhat true, 2 = certainly true). SDQ is a useful screening tool for identifying mental health difficulties and strengths in children. Total difficulties scores, reflecting emotional, and behavioral difficulties, were calculated by summing the first four of the subscale scores, with higher scores representing greater difficulties. The prosocial behavior scores were calculated by the last subscale, with lower scores indicating less prosocial behavior. Four degrees of total difficulties were defined according to total difficulties subscale scores (“Normal,” score 0–13; “Borderline,” score 14–16; “Moderately abnormal,” score 17–19; and “Prominently abnormal,” score 20–40). Four degrees of prosocial behavior were classified based on prosocial behavior subscale scores (“Normal,” score 8–10; “Borderline,” score 7; “Moderately abnormal,” score 6; and “Prominently abnormal,” score 0–5) (Du et al., 2008). The Chinese version of parent-reported SDQ showed strong internal consistency (Cronbach’s alpha coefficients for the total difficulties scale and the prosocial behavior subscale were 0.81 and 0.87, respectively), and moderate test-retest reliability (Pearson’s correlation coefficients for the total difficulties scale and the prosocial behavior subscale were 0.71 and 0.61, respectively, over a 2-month interval) (Yao et al., 2009). It also had a good parallel validity with Conners Parent Questionnaire in behavioral and emotional problems of Chinese children and adolescents (Kou et al., 2005).

### Statistical Analysis

Continuous variables were represented as mean (standard deviation, SD), while categorical variables as frequency (percentage, %). Chi-square test was utilized to compare the prevalence of normal, borderline, moderately abnormal, and prominently abnormal scores for total difficulties and prosocial behavior, respectively, among children with different characteristics. Grade (1–5) and age (5–13 years) were highly correlated (Spearman correlation coefficients: 0.963), and grade variable was selected for further analysis because there were several missing values for age. Ordinal logistic regression analyses were used to evaluate the impact of psychological stressors, daily activities, and social interactions on total difficulties and prosocial behavior according to the four categories of SDQ scores, based on the crude model (Model 1) and the adjusted model (Model 2). Model 2 adjusted for child characteristics (grade, gender, school level, child medical conditions, knowledge levels on COVID-19, and precaution levels on COVID-19) and family characteristics (including area, number of household rooms, economic status, education of caregiver, caregiver medical conditions, and caregiver–child relationship). The Brant test showed that the proportional odds assumption was met. The results were presented as odds ratios (ORs) and 95% confidence intervals (CIs). Interactions between psychological stressors and sociodemographic characteristics were tested. Stratified analyses were conducted according to grade, gender, school level, area, and caregiver–child relationship with interaction terms in the regression models to further observe the associations of total difficulties and prosocial behavior with the number of psychological stressors. The level of statistical significance was defined as α = 0.05 of two-side probability. All analyses were performed by R program (version 4.0.4, R Foundation for Statistical Computing, Vienna, Austria), and all figures were performed by GraphPad Prism software (version 7, GraphPad Prism, CA, United States).

## Results

### Distributions of Total Difficulties Scores and Prosocial Behavior Scores

Of those eligible participants (*n* = 6017), the mean score of SDQ on total difficulties and prosocial behavior was 8.08 (±4.51) and 7.59 (±1.99), respectively. The prevalence of borderline, moderately abnormal and prominently abnormal scores were 7.16% (*n* = 431), 3.34% (*n* = 201), and 1.96% (*n* = 118) for total difficulties and 13.83% (*n* = 832), 13.45% (*n* = 809), and 17.85% (*n* = 1074) for prosocial behavior, respectively (Table 1). Boys, children with medical conditions (acute or chronic illnesses or mental disorders), children with less knowledge and precautionary measures toward COVID-19, fewer household rooms, lower family economic status, lower caregiver education level and poor caregiver–child relationship were more likely to have both abnormal total difficulties scores and prosocial behavior scores, compared with the counterparts (*P* < 0.05). In addition, children in Grade 5, from non-key school, from Taizhou may have less prosocial behavior than their counterparts (*P* < 0.05). Parents having medical conditions potentially increased their child’s total difficulties (*P* < 0.05).

### Status of Psychological Stressors, Daily Activities, and Social Interactions

When schools reopened, most of children (62.51%, *n* = 3761) were afraid of, to varying degrees, lagging behind in school lessons. Over 30% of participants were, to some extent, afraid of going to school (47.43%, *n* = 2854), keeping social distance with others (45.75%, *n* = 2753), inadaptation to study and life styles (37.86%, *n* = 2278), and changes in relationship with schoolmates (30.8%, *n* = 1853). Above 27% (*n* = 1625) of participants had online learning and 10.42% (*n* = 627) did homework more than 3 h daily in the last week. The majority did physical exercises (93.77%, *n* = 5462) and had some types of communication with friends or relatives in the last week, including telephone or video calling (75.93%, *n* = 4569), text messaging or social media communication (68.22%, *n* = 4105), and face-to-face meeting (59.73%, *n* = 3594). More than half of children (54.98%, *n* = 3308) played computer games in the past week. All these psychological stressors, daily activities, and social interactions, except online learning, had an impact on total difficulties and prosocial behavior scores (*P* < 0.001) (Table 2).

### Associations Between Psychological Stressors, Daily Activities, Social Interactions, and Strengths and Difficulties Questionnaire Scores

Results of ordinal logistic regression analyses were shown in Table 3. Compared with children without psychological stressors, those with concerns had a significantly higher risk of being in a worse category of mental health status after schools reopened. The associations between psychological stressors and total difficulties were progressively stronger in children with increased degrees of concerns, with the maximum adjusted OR of 7.90 (95% CI 3.33–18.75) observed in those who were definitely afraid of inadaptation to study and life styles. The associations for prosocial behavior were more moderate, with the maximum adjusted OR of 4.54 (95% CI 2.80–7.34) observed in those who were definitely afraid of going to school. Time used in home work and computer games was positively related to mental health problems, while frequency of communication with others was negatively related. Children who did physical exercises at least half an hour daily in the last week were less likely to be in a worse category of total difficulties (OR = 0.27, 95% CI 0.21–0.36) and of prosocial behavior (OR = 0.41, 95% CI 0.34–0.51), compared with those who did not.

**TABLE 3:** Associations of total difficulties and prosocial behavior with psychological stressors, daily activities, and social interactions by ordinal logistic regression models.

	**ORs (95% CI) for risks of psychological problems**			
	**Total difficulties**		**Prosocial behavior**	
	**Model 1^c^**	**Model 2^c^**	**Model 1^c^**	**Model 2^c^**
**Psychological stressors^a^**				
**Going to school**				
Completely not	1.00	1.00	1.00	1.00
Slightly	2.46 (2.05–2.95)	2.07 (1.71–2.50)	2.50 (2.25–2.78)	2.01 (1.80–2.25)
Moderately	5.86 (4.70–7.30)	4.18 (3.32–5.28)	4.73 (4.02–5.56)	3.32 (2.80–3.92)
Definitely	9.10 (5.53–14.99)	5.38 (3.14–9.21)	6.86 (4.30–10.93)	4.54 (2.80–7.34)
**Lagging in school lessons**				
Completely not	1.00	1.00	1.00	1.00
Slightly	1.83 (1.51–2.22)	1.73 (1.41–2.12)	1.48 (1.33–1.65)	1.29 (1.15–1.45)
Moderately	3.11 (2.46–3.92)	2.79 (2.17–3.59)	1.6 (1.38–1.87)	1.35 (1.15–1.59)
Definitely	3.99 (2.99–5.32)	3.98 (2.91–5.46)	1.07 (0.86–1.34)	0.93 (0.73–1.17)
**Inadaptation to study and life styles**				
Completely not	1.00	1.00	1.00	1.00
Slightly	3.55 (3.01–4.19)	3.12 (2.63–3.71)	2.44 (2.2–2.7)	2.01 (1.81–2.23)
Moderately	8.03 (5.73–11.25)	6.25 (4.39–8.89)	2.46 (1.84–3.29)	1.80 (1.33–2.43)
Definitely	12.28 (5.4–27.92)	7.90 (3.33–18.75)	4.05 (1.88–8.71)	2.54 (1.15–5.59)
**Keeping social distance with others**				
Completely not	1.00	1.00	1.00	1.00
Slightly	2.03 (1.72–2.39)	1.88 (1.58–2.24)	1.63 (1.47–1.81)	1.49 (1.34–1.66)
Moderately	2.32 (1.75–3.09)	2.15 (1.59–2.90)	1.53 (1.26–1.86)	1.46 (1.18–1.78)
Definitely	3.06 (2.08–4.51)	3.10 (2.05–4.68)	0.78 (0.56–1.07)	0.75 (0.53–1.05)
**Changes in relationship with schoolmates**				
Completely not	1.00	1.00	1.00	1.00
Slightly	3.16 (2.68–3.74)	2.87 (2.41–3.41)	1.97 (1.77–2.2)	1.70 (1.51–1.91)
Moderately	4.97 (3.7–6.68)	4.25 (3.11–5.81)	1.72 (1.36–2.19)	1.32 (1.03–1.69)
Definitely	3.31 (2.15–5.1)	3.25 (2.06–5.12)	0.94 (0.66–1.35)	0.92 (0.64–1.34)
**Daily activities in the last week^c^,^b^**				
**Online learning**				
<1 h/day	1.00	1.00	1.00	1.00
1–3 h/day	1.2 (0.99–1.45)	1.13 (0.93–1.38)	1.04 (0.92–1.17)	1.01 (0.89–1.14)
>3 h/day	1.09 (0.91–1.31)	1.09 (0.89–1.34)	0.96 (0.85–1.08)	1.05 (0.93–1.19)
**Homework**				
<1 h/day	1.00	1.00	1.00	1.00
1–3 h/day	1.34 (1.09–1.64)	1.34 (1.08–1.65)	1.13 (1.01–1.27)	1.10 (0.98–1.24)
>3 h/day	3.72 (2.89–4.79)	3.34 (2.56–4.35)	1.64 (1.37–1.95)	1.41 (1.17–1.69)
**Computer games**				
None	1.00	1.00	1.00	1.00
=1 time/day	1.36 (1.13–1.62)	1.23 (1.02–1.49)	1.31 (1.18–1.46)	1.18 (1.06–1.33)
>1 time/day	3.51 (2.87–4.29)	2.89 (2.33–3.58)	2.07 (1.8–2.39)	1.67 (1.44–1.95)
**Physical exercise**				
None	1.00	1.00	1.00	1.00
=30 min/day	0.32 (0.25–0.4)	0.43 (0.34–0.56)	0.54 (0.44–0.66)	0.69 (0.57–0.86)
>30 min/day	0.17 (0.13–0.22)	0.27 (0.21–0.36)	0.27 (0.22–0.33)	0.41 (0.34–0.51)
**Social interactions in the last week**				
**Telephone or video calling communication**				
None	1.00	1.00	1.00	1.00
=1 time/day	0.61 (0.51–0.72)	0.65 (0.54–0.78)	0.69 (0.62–0.77)	0.7 (0.62–0.79)
>1 time/day	0.65 (0.51–0.83)	0.79 (0.61–1.02)	0.56 (0.48–0.66)	0.66 (0.56–0.79)
**Text messaging or social media communication**				
None	1.00	1.00	1.00	1.00
=1 time/day	0.66 (0.55–0.78)	0.71 (0.6–0.85)	0.75 (0.67–0.84)	0.78 (0.69–0.87)
>1 time/day	0.66 (0.53–0.82)	0.77 (0.62–0.97)	0.64 (0.55–0.73)	0.71 (0.61–0.82)
**Meeting with friends or relatives**				
None	1.00	1.00	1.00	1.00
=1 time/day	0.79 (0.67–0.94)	0.81 (0.68–0.97)	0.88 (0.79–0.98)	0.88 (0.79–0.98)
>1 time/day	1.22 (0.98–1.53)	1.26 (0.99–1.58)	0.75 (0.64–0.88)	0.77 (0.66–0.91)

*^*a*^The degree to which a child was afraid of each situation after school reopened.*

*^*b*^The length of time/frequency a child conducted each activity in the last week.*

*^*c*^Model 1: unadjusted; Model 2: adjusted for child characteristics (including grade, gender, school level, child medical conditions, knowledge levels on COVID-19, and precaution levels on COVID-19) and family characteristics (including area, number of household rooms, economic status, education of caregiver, caregiver medical conditions, and caregiver–child relationship).*

### Associations Between Psychological Stressors and Strengths and Difficulties Questionnaire Scores Stratified by Sociodemographic Characteristics

**FIGURE 2:**
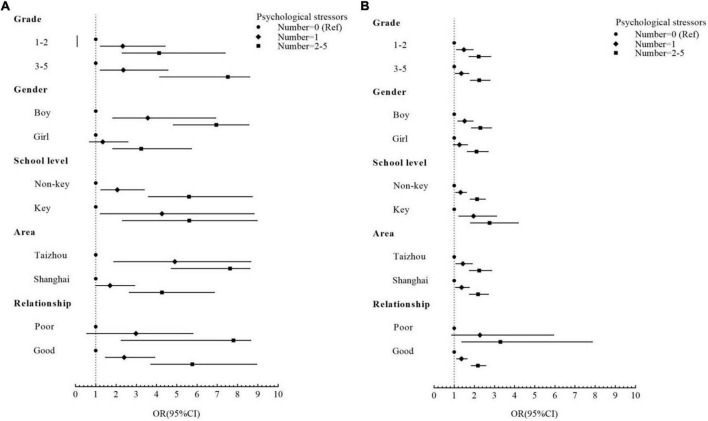
Odds ratio (ORs) and 95% confidence intervals (95% CIs) for risk of **(A)** total difficulties and **(B)** prosocial behavior by the effects of the number of psychological stressors after being stratified by grade, gender, school level, area, and caregiver–child relationship based on Model 2.

There was an accumulative effect of the number of psychological stressors on total difficulties and prosocial behavior. The magnitude of risk to total difficulties varied in children with different characteristics in stratified analyses (Figure 2). Compared with children without psychological stressors, those with two or more stressors were more likely to be in a worse category of total difficulties, and the results were more evident in middle-high grade (Grade 3–5) students (OR = 7.52, 95% CI 4.16–8.61), boys (OR = 6.95, 95% CI 4.83–8.55), those who lived in Taizhou (OR = 7.62, 95% CI 4.72–8.61) and with poor caregiver–child relationship (OR = 7.79, 95% CI 2.26–8.65) (Figure 2A). Key school students and those with poor caregiver–child relationship were more vulnerable to the effects of psychological stressors on prosocial behavior, with the corresponding adjusted ORs of 2.75 (95% CI 1.81–4.18) and 3.29 (95% CI 1.38–7.86), respectively (Figure 2B).

## Discussion

In this current study, we observed that the prevalence of borderline to prominently abnormal scores was 12.46% for total difficulties and 45.12% for prosocial behavior, respectively, among primary school students after schools reopened. In addition, the prevalence of prominently abnormal scores was 1.96% for total difficulties and 17.85% for prosocial behavior. Psychological stressors caused by school reopening and social distancing measures, and longer time involved in homework and computer games were related to increased risks of mental health problems. In contrast, more physical exercise and more social interactions may have beneficial effects on mental health. Moreover, boys, middle-high grade (Grade 3–5) or key school students, those living in Taizhou and those with poor caregiver–child relationship were more sensitive to the effect of psychological stressors on mental health status.

The mean score of total difficulties was 8.08 (±4.51) among 6017 students (aged 5–13 years) in this study, which was lower than that of 9.63 (±4.79) among the 1264 students (aged 7–12 years) in a study conducted in Hubei Province, China, between February 25 and March 8, 2020 (the total difficulties were measured using the Chinese version of parent-reported SDQ for grades 2–3 and the Chinese version of self-reported SDQ for grades 4–6) (Liu et al., 2020), and much lower than that of 11.59 (±5.57) among 17,029 children (aged 6–12 years) in a study conducted in Hong Kong, China, in late March 2020 (using the Chinese version of parent-reported SDQ) (Tso et al., 2020). These findings suggested that, compared with children under school closure and home confinement, those who returned to school may experience less mental health difficulties. School routine consists an important coping mechanism for children to maintain mental health, allowing them to have a regular schedule, interact with their peers, and focus on achieving meaningful goals (Lee, 2020). In addition, we did not observe a higher mean score of total difficulties in the current study than that of children (10.89 ± 4.84, aged 3–10 years) in Shanghai prior to COVID-19 (using the Chinese version of parent-reported SDQ) (Du et al., 2008). There is a cumulative risk theory that short term exposure to trauma and adversities may not obviously affect children’s emotional and behavioral problems, but make them more vulnerable to disadvantages experienced at a later stage (Gutman et al., 2019). Therefore, longitudinal research on children’s mental health is necessary to further understand the long-term effect of the COVID-19 pandemic.

Prosocial behavior typically refers to a large class of voluntary behaviors that share the common intention to benefit another, by helping, sharing, comforting, informing, and cooperating (Dunfield and Kuhlmeier, 2013). In this study, 17.85% children reported having prominently abnormal prosocial behavior scores, the prevalence of which was higher than that of children in Hubei Province during the early period of home quarantine (10.3%) (Liu et al., 2020), and that of children in Shanghai prior to COVID-19 (9.1%) (Du et al., 2008). However, prosocial score ≤4 was used as a cutoff score for prosocial problems in the above studies, while a score ≤5 was considered as prominently abnormal in this study according to a newer 4-band categorization recommended by the inventors of SDQ (Youthinmind, 2021). The mean score of prosocial behavior in our study (7.59 ± 1.99) was comparable to theirs during home confinement (7.24 ± 2.19) (Liu et al., 2020), but higher than that of Chinese children before the pandemic (5.7 ± 2.2–6.6 ± 2.1) (using the Chinese version of parent-reported SDQ) (Gao et al., 2013). It is noteworthy that the prevalence of prosocial behavior problems was high in children during the pandemic period when a set of new norms were created and unique stressors were faced by students (McDonald, 2020).

In this study, children who experienced psychological stressors and who were involved in longtime homework or computer games were less likely to present prosocial behavior. The consequences of 3- to 4-month gap in normal schooling might be extensive with adverse impacts on learning motivation, self-control, and social competence (Fontenelle-Tereshchuk, 1984). We found that more than 30% of children were afraid of inadaptation to social activities after schools reopened, such as keeping social distance with others (45.75%) and changes in relationship with schoolmates (30.8%). Some children might be unwilling to be close to others due to concerns over the spread of coronavirus and thus expressed less prosocial behaviors. Chronic stress has also been reported to reduce social motivation and social interactions, while social stressors such as social isolation may increase aggressiveness (Sandi and Haller, 2015). Although these students have returned to the campus, some children might fail to complete the transition to normal lives. Long exposure to digital screens and addiction to computer games may cause reduced vision and sedentary lifestyle (Mittal et al., 2020), leading to physical and mental health problems (Lemola et al., 2015; Alhassan et al., 2018). Therefore, the chronic stress children experienced and the long-time isolated lives before the schools reopened were detrimental to their prosocial behaviors. In addition, our findings showed that physical exercises and social interactions were protective factors for prosocial behavior. Consistent with our study, a research conducted in the United States found a significant relationship between prosocial behavior and moderate-intensity physical exercise (Moore et al., 2020). In terms of social interactions, Barry and Wentzel reported that the quantity and quality of social contact moderated relations between a friend’s prosocial behavior and an individual’s prosocial goal pursuit, which in turn, affected the individual’s prosocial behavior (Barry and Wentzel, 2006).

The magnitude of the risk of psychological stressors to emotional and behavioral problems was different across participants, and was larger in boys, middle-high grade or key school students, children with poor caregiver–child relationship and living in Taizhou. During the long-time period of schooling at home, children had limited interaction with teachers and classmates, and largely relied on their parents or other main caregivers for emotional and academic support (Fontenelle-Tereshchuk, 1984). Parenthood is a demanding role, particularly under the influence of COVID-19 pandemic. Parents had to manage multiple tasks and spend much more time with their children, thus increasing the risk of conflict between them. A study conducted in Taizhou reported that when students had study problems due to school closures, those with poor parent–child relationship were particularly vulnerable to depressive symptoms (Wang et al., 2021). On the contrary, harmonious parent–child relationship, especially parental supportive behaviors and responsive parenting, tend to alleviate the effects of stressors on children’s mental health and promote their prosocial behavior (Eisenberg et al., 2019; Tang et al., 2020). Boys in childhood may have more hyperactivity issues, more conduct problems and poorer self-discipline than girls (Gao et al., 2013), and thus they may experience higher levels of stress and anxiety after returning to campus (Duckworth and Seligman, 2006). Students in higher grade and key schools tend to have more academic pressure and achievement expectation than the others. Schooling at home for a long time may take a toll on their study progress and is especially a stressor to these students (Tang et al., 2020). The safety measures on campus for pandemic control after school reopening, to some degree, restricted physical activities and social interactions for children, and included repeated nucleic acid testing which may lead to a new round of psychological anxiety and emotional distress (Wu, 2020). The time of school reopening in Taizhou was approximately 1 month earlier than in Shanghai, and thus children living in Taizhou might be more greatly affected by stricter safety measures. In the current study, 85.7% children in Taizhou experienced psychological stressors, while 82.5% in Shanghai. In addition, the percentages of boys (56.7 vs. 43.3%, *P* = 0.003) and those who studied in key schools (30.9 vs. 8.1%, *P* < 0.001) were significantly larger in Taizhou than those in Shanghai, which may in part explain the regional difference in the results.

To our knowledge, this is the first study to evaluate the mental health status of primary school students after schools reopened during the COVID-19 post-pandemic in China, with a large sample size and a hot issue warranted to be paid more attention in the current society. Understanding children’s reactions and resilience to great changes in their lives in difficult times is essential to properly address their needs and promote their mental well-being. Our study provides a preliminary understanding of behavioral and emotional problems, as well as their related factors of primary school students in two regions in east China after a period of school reopening. It is noteworthy that school reopening could be a psychological stressor *per se*. Students need to face inadaptation to lifestyle changes again and social distance requirements. School psychologists need to increase their level of service to support students’ transition back into the school building and address emotional and behavioral problems.

The results of this study should be interpreted with a number of limitations. First, this was a cross-sectional study, so we were unable to collect detailed information on the mental health status of children before schools reopened. However, we had a question of child medical conditions which included psychiatric illnesses, and we adjusted for this variable in the regression models. Future longitudinal studies will allow a better understanding of the long-term effects of the COVID-19 outbreak on children’s psychological adjustment. Second, subjects in this study were residing in two regions in east China, and the findings may not be fully applicable to children living in other areas. Third, the information of children was collected from their caregivers, and the potential bias inherent in this study should be noted. Although our study has some limitations, the results could help to inform future research to enable children’s mental well-being and minimize their distress. More focused research should be performed directly on testimony to children’s experience during the pandemic apart from indirect information collected from their caregivers. Qualitative research could be considered to fully understand children’s feelings and identify approaches in which the negative impact of COVID-19 might be alleviated. Future research should also endeavor to ascertain the longer-term consequences of the pandemic on psychological well-being for children, determine the mechanisms which explain the prevalence of emotional and behavioral difficulties, and develop novel interventions to improve mental health such as those based on altruism and prosocial behavior (Holmes et al., 2020).

The present study has some important implications. The Chinese government, community, school, and family need to provide more effective support for children after school reopened. The government needs to recognize the public health challenge of children’s mental health, ensure multifaceted and coordinated actions between schools, local health professionals, and other organizations, and provide sufficient funding to support relevant research and practice. Communities can collaborate with mental health professionals in providing online and offline psychological services for high-risk individuals, and specialized psychoeducation for children and their caregivers to improve coping strategies and resilience for stressful life changes and negative situations. Schools should try to alleviate students’ academic pressure by reducing non-essential homework and class ranking, and set up some engaging lessons related to emotion management, positive psychology, and so on. Teachers should pay more attention to students’ emotional and behavioral problems, and refer students with difficulties to school psychologists. Caregivers need to provide consistent and sensitive care as well as age-appropriate information about the pandemic, and opportunities for kids to talk about their concerns (Dalton et al., 2020). Furthermore, caregivers and teachers should foster children’s prosocial tendency, as helping others might distract an individual from stressors and increase one’s sense of meaning, purpose, and self-efficacy (Raposa et al., 2016). Prosocial behavior has also been identified as an important contributor to resilience for kids experiencing hardship (Daud et al., 2008). Evidence suggested that keeping in touch with friends and family can ease mental distresses (Feng and Astell-Burt, 2016), and the similar results were also indicated in this study. During the pandemic, adults should help children find effective approaches to staying connected such as online video chats. It is also important to encourage children to engage in restorative activities that help them self-regulate, e.g., physical exercise and outdoor activities which may lead to reduced anxiety, improvements in self-esteem, and cognitive performance (Biddle and Asare, 2011).

## Conclusion

Emotional and behavioral difficulties, especially prosocial behavior problems, existed in primary school children after schools reopened, which may be aggravated by psychological stressors, longtime home work, and excessive computer games. Conversely, more physical exercise and social interactions may alleviate these mental health problems. Boys, middle-high grade or key school students, and those with poor caregiver–child relationship may be especially vulnerable to psychological stressors during their transition back to school.

## Data Availability Statement

The datasets for this manuscript will be made available upon request pending, further inquiries can be directed to the corresponding author CF, fcw@fudan.edu.cn.

## Ethics Statement

The studies involving human participants were reviewed and approved by the Ethical Review Board of School of Public Health of Fudan University. Written informed consent to participate in this study was provided by the participants’ legal guardian/next of kin.

## Author Contributions

CF, XG, XC, and HJL designed the study. HW, HJL, and HBL were involved in data collection and assembly. JW and YW analyzed and interpreted the data, and drafted the manuscript as co-first authors. CF and XG reviewed and revised the manuscript. All authors contributed to manuscript writing and approved the final manuscript.

## Conflict of Interest

The authors declare that the research was conducted in the absence of any commercial or financial relationships that could be construed as a potential conflict of interest.

## Publisher’s Note

All claims expressed in this article are solely those of the authors and do not necessarily represent those of their affiliated organizations, or those of the publisher, the editors and the reviewers. Any product that may be evaluated in this article, or claim that may be made by its manufacturer, is not guaranteed or endorsed by the publisher.

## Footnotes

1
https://www.wjx.cn
